# Author Correction: Genome-based *Salmonella* serotyping as the new gold standard

**DOI:** 10.1038/s41598-020-67917-3

**Published:** 2020-06-26

**Authors:** Sangeeta Banerji, Sandra Simon, Andreas Tille, Angelika Fruth, Antje Flieger

**Affiliations:** 1 Division of Enteropathogenic Bacteria and Legionella (FG11)/National Reference Centre for Salmonella and Other Bacterial Enteric Pathogens, Robert Koch-Institute, Burgstrasse 37, 38855 Wernigerode, Germany; 2 Department for Infectious Disease Epidemiology (FG31), Robert Koch-Institute, Burgstrasse 37, 38855 Wernigerode, Germany

Correction to: *Scientific Reports* 10.1038/s41598-020-61254-1, published online 9 March 2020

This Article contains errors in Table 1, where the data for the rows 'Paratyphi C' and 'Poano' have been omitted. The correct Table 1 appears below.

**Table 1:** Overview of serotype prediction with SeqSero and MLST.

Serotype	Sequenced isolates	Correlation			Ambiguous			Prediction failure			Miscorrelation		
		Seq-Sero	MLST	Seq-Sero + MLST	Seq-Sero	MLST	Seq-Sero + MLST	Seq-Sero	MLST	Seq-Sero + MLST	Seq-Sero	MLST	Seq-Sero + MLST
Agona	3	3	3	3	0	0	0	0	0	0	0	0	0
Choleraesuis	33	0	33	33	30 or Typhisuis or Paratyphi C	0	0	3	0	0	0	0	0
Choleraesuis monophasic	3	0	0	0	0	0	0	0	0	0	3	3	3
Derby	55	55	55	55	0	0	0	0	0	0	0	0	0
11:z41:e,n,z15 (novel serovar)	10	10	10	10	0	0	0	0	0	0	0	0	0
Enteritidis	115	115	115	115	0	0	0	0	0	0	0	0	0
Infantis	50	49	50	50	0	0	0	1	0	0	0	0	0
Kentucky	7	7	7	7	0	0	0	0	0	0	0	0	0
Kintambo	3	0	3	3	3 or Washington	0	0	0	0	0	0	0	0
Kottbus	12	0	12	12	12 or Ferruch	0	0	0	0	0	0	0	0
Mbandaka	15	15	15	15	0	0	0	0	0	0	0	0	0
Mikawasima	10	10	10	10	0	0	0	0	0	0	0	0	0
Muenchen	25	0	25	25	25 or Virginia	0	0	0	0	0	0	0	0
Paratyphi B	6	6	6	6	0	0	0	0	0	0	0	0	0
Paratyphi B monophasic	1	1	1	1	0	0	0	0	0	0	0	0	0
Paratyphi C	2	0	2	2	2 or Cholerae-suis or Typhisuis	0	0	0	0	0	0	0	0
Poano	2	2	2	2	0	0	0	0	0	0	0	0	0
Strathcona	2	2	2	2	0	0	0	0	0	0	0	0	0
Stourbridge	14	14	14	14	0	0	0	0	0	0	0	0	0
Sundsvall	1	0	1	1	1 or Soahanina or Sundvall	0	0	0	0	0	0	0	0
Typhi	74	74	74	74	0	0	0	0	0	0	0	0	0
Typhimurium biphasic	52	52	32	52	0	0	0	0	0	0	0	20	0
Typhimurium monophasic	19	17	17	19	0	0	0	0	0	0	2	2	0
Serologically rough	6	5	6	6	0	0	0	1	0	0	0	0	0
Total number	520	437	495	517	73	0	0	5	0	0	5	25	3
Total (%)	100.0	84.0	95.2	99.4	14.0	0	0	1.0	0	0	1.0	4.8	0.6
Overall (%)	100.0	98.0	95.2	99.4	–	–	–	1.0	0	0	1.0	4.8	0.6

Serotype was first determined by classical serotyping. Whole genome sequences were then analyzed with SeqSero or MLST. Correlation means that the predicted serotype was the same as the classically determined serovar. Ambiguous means that the correct serotype was listed among others. Prediction failure means that no complete antigenic formula was derived. Miscorrelation means that a wrong antigenic formula was derived. Overall (%) is the sum of Ambiguous (%) and Correlation (%). Final results are shown, i.e. after resequencing if data quality was not met.

The Supplementary file that accompanies this Article also contains an error in which Supplementary Table S1 is missing. The correct Supplementary Table S1 appears below as Table 2.

**Table 2:** Overview of NRC dataset for the study Banerji et al.: ‘Genome-based Salmonella serotyping as the new Gold Standard’.

ENA run number	Source	Year of isolation	Country of isolation	Serovar	ENA project
ERR3263618	Human	2009	Germany	S. Infantis	PRJEB30317
ERR3263619	Human	2009	Germany	S. Infantis	PRJEB30317
ERR3263620	Human	2009	Germany	S. Infantis	PRJEB30317
ERR3263621	Human	2001	Germany	S. Sundsvall	PRJEB30317
ERR3263622	Meat	2004	Germany	S. Derby	PRJEB30317
ERR3263623	Human	2006	Germany	S. Typhimurium mono	PRJEB30317
ERR3263624	Human	2007	Germany	S. Kintambo	PRJEB30317
ERR3263625	Human	2007	Germany	S. Derby	PRJEB30317
ERR3263626	Human	2007	Germany	S. Poano	PRJEB30317
ERR3263627	Human	2007	Germany	S. Derby	PRJEB30317
ERR3263628	Human	2007	Germany	S. Derby	PRJEB30317
ERR3263629	Human	2007	Germany	S. Derby	PRJEB30317
ERR3263630	Human	2008	Germany	S. Derby	PRJEB30317
ERR3263631	Human	2009	Germany	S. Kintambo	PRJEB30317
ERR3263632	Human	2011	Germany	S. Kintambo	PRJEB30317
ERR3263633	Human	2011	Germany	S. Poano	PRJEB30317
ERR3263634	Meat	2012	Germany	S. Derby	PRJEB30317
ERR3263635	Human	2012	Germany	S. Derby	PRJEB30317
ERR3263636	Human	2012	Germany	S. Derby	PRJEB30317
ERR3263637	Human	2012	Germany	S. Derby	PRJEB30317
ERR3263638	Poultry	2012	Germany	S. Derby	PRJEB30317
ERR3263639	Human	2012	Germany	S. Derby	PRJEB30317
ERR3263640	Human	2013	Germany	S. Infantis	PRJEB30317
ERR3263641	Human	2013	Germany	S. Infantis	PRJEB30317
ERR3263642	Cattle	2013	Germany	S. Infantis	PRJEB30317
ERR3263643	Human	2013	Germany	S. Derby	PRJEB30317
ERR3263644	Human	2013	Germany	S. Muenchen	PRJEB30317
ERR3263645	Human	2013	Germany	S. Muenchen	PRJEB30317
ERR3263646	Meat	2013	Germany	S. Muenchen	PRJEB30317
ERR3263647	Human	2013	Germany	S. Muenchen	PRJEB30317
ERR3263648	Human	2013	Germany	S. Muenchen	PRJEB30317
ERR3263649	Pig	2013	Germany	S. Muenchen	PRJEB30317
ERR3263650	Pig	2013	Germany	S. Muenchen	PRJEB30317
ERR3263651	Human	2013	Germany	S. Derby	PRJEB30317
ERR3263652	Human	2013	Germany	S. Derby	PRJEB30317
ERR3263653	Human	2013	Germany	S. Derby	PRJEB30317
ERR3263654	Human	2013	Germany	S. Derby	PRJEB30317
ERR3263655	Human	2013	Germany	S. Derby	PRJEB30317
ERR3263656	Human	2014	Germany	S. Derby	PRJEB30317
ERR3263657	Human	2014	Germany	S. Derby	PRJEB30317
ERR3263658	Human	2014	Germany	S. Derby	PRJEB30317
ERR3263659	Human	2014	Germany	S. Derby	PRJEB30317
ERR3263660	Human	2014	Germany	S. Derby	PRJEB30317
ERR3263661	Human	2014	Germany	S. Derby	PRJEB30317
ERR3263662	Human	2014	Germany	S. Derby	PRJEB30317
ERR3263663	Human	2014	Germany	S. Derby	PRJEB30317
ERR3263664	Human	2014	Germany	S. Derby	PRJEB30317
ERR3263665	Human	2014	Germany	S. Derby	PRJEB30317
ERR3263666	Human	2014	Germany	S. Derby	PRJEB30317
ERR3263667	Human	2014	Germany	S. Derby	PRJEB30317
ERR3263668	Human	2014	Germany	S. Derby	PRJEB30317
ERR3263669	Human	2014	Germany	S. Derby	PRJEB30317
ERR3263670	Human	2014	Germany	S. Muenchen	PRJEB30317
ERR3263671	Human	2014	Germany	S. Derby	PRJEB30317
ERR3263672	Human	2014	Germany	S. Derby	PRJEB30317
ERR3263673	Human	2014	Germany	S. Derby	PRJEB30317
ERR3263674	Human	2014	Germany	S. Derby	PRJEB30317
ERR3263675	Human	2014	Germany	S. Derby	PRJEB30317
ERR3263676	Human	2014	Germany	S. Derby	PRJEB30317
ERR3263677	Human	2014	Germany	S. Derby	PRJEB30317
ERR3263678	Human	2014	Germany	S. Derby	PRJEB30317
ERR3263679	Human	2014	Germany	S. Derby	PRJEB30317
ERR3263680	Human	2014	Germany	S. Muenchen	PRJEB30317
ERR3263681	Human	2014	Germany	S. Derby	PRJEB30317
ERR3263682	Human	2014	Germany	S. Derby	PRJEB30317
ERR3263683	Human	2014	Germany	S. Derby	PRJEB30317
ERR3263684	Human	2014	Germany	S. Derby	PRJEB30317
ERR3263685	Human	2014	Germany	S. Derby	PRJEB30317
ERR3263686	Human	2014	Germany	S. Derby	PRJEB30317
ERR3263687	Human	2014	Germany	S. Derby	PRJEB30317
ERR3263688	Human	2014	Germany	S. Derby	PRJEB30317
ERR3263689	Human	2014	Germany	S. Derby	PRJEB30317
ERR3263690	Human	2014	Germany	S. Muenchen	PRJEB30317
ERR3263691	Human	2014	Germany	S. Muenchen	PRJEB30317
ERR3263692	Human	2014	Germany	S. Muenchen	PRJEB30317
ERR3263693	Human	2014	Germany	S. Muenchen	PRJEB30317
ERR3263694	Human	2014	Germany	S. Muenchen	PRJEB30317
ERR3263695	Human	2014	Germany	S. Muenchen	PRJEB30317
ERR3263696	Human	2014	Germany	S. Muenchen	PRJEB30317
ERR3263697	Human	2014	Germany	S. Muenchen	PRJEB30317
ERR3263698	Meat	2014	Germany	S. Muenchen	PRJEB30317
ERR3263699	Human	2014	Germany	S. Muenchen	PRJEB30317
ERR3263700	Meat	2014	Germany	S. Muenchen	PRJEB30317
ERR3263701	Meat	2014	Germany	S. Muenchen	PRJEB30317
ERR3263702	Human	2014	Germany	S. Muenchen	PRJEB30317
ERR3263703	Pig	2014	Germany	S. Muenchen	PRJEB30317
ERR3263704	Pig	2014	Germany	S. Muenchen	PRJEB30317
ERR3263705	Human	2014	Germany	S. Muenchen	PRJEB30317
ERR3263706	Human	2014	Germany	S. Infantis	PRJEB30317
ERR3263707	Human	2014	Germany	S. Enteritidis	PRJEB30317
ERR3263708	Human	2014	Germany	S. Enteritidis	PRJEB30317
ERR3263709	Human	2014	Germany	S. Enteritidis	PRJEB30317
ERR3263710	Human	2014	Germany	S. Enteritidis	PRJEB30317
ERR3263711	Human	2014	Germany	S. Enteritidis	PRJEB30317
ERR3263712	Human	2014	Germany	S. Enteritidis	PRJEB30317
ERR3263713	Human	2014	Germany	S. Enteritidis	PRJEB30317
ERR3263714	Human	2014	Germany	S. Enteritidis	PRJEB30317
ERR3263715	Human	2014	Germany	S. Enteritidis	PRJEB30317
ERR3263716	Human	2014	Germany	S. Enteritidis	PRJEB30317
ERR3263717	Human	2014	Germany	S. Enteritidis	PRJEB30317
ERR3263718	Human	2014	Germany	S. Enteritidis	PRJEB30317
ERR3263719	Human	2014	Germany	S. Enteritidis	PRJEB30317
ERR3263720	Human	2014	Germany	S. Enteritidis	PRJEB30317
ERR3263721	Human	2014	Germany	S. Enteritidis	PRJEB30317
ERR3263722	Human	2015	Germany	S. Typhi	PRJEB30317
ERR3263723	Human	2015	Germany	S. Enteritidis	PRJEB30317
ERR3263724	Human	2015	Germany	S. Typhi	PRJEB30317
ERR3263725	Human	2015	Germany	S. Enteritidis	PRJEB30317
ERR3263726	Human	2015	Germany	S. Enteritidis	PRJEB30317
ERR3263727	Human	2015	Germany	S. Enteritidis	PRJEB30317
ERR3263728	Human	2015	Germany	S. Enteritidis	PRJEB30317
ERR3263729	Human	2015	Germany	S. Typhi	PRJEB30317
ERR3263730	Human	2015	Germany	S. Typhi	PRJEB30317
ERR3263731	Human	2015	Germany	S. Enteritidis	PRJEB30317
ERR3263732	Human	2015	Germany	S. Typhi	PRJEB30317
ERR3263733	Human	2015	Germany	S. Typhi	PRJEB30317
ERR3263734	Human	2015	Germany	S. Typhi	PRJEB30317
ERR3263735	Human	2015	Germany	S. Typhi	PRJEB30317
ERR3263736	Human	2015	Germany	S. Typhi	PRJEB30317
ERR3263737	Human	2015	Germany	S. Typhi	PRJEB30317
ERR3263738	Not provided	2015	Germany	S. Typhi	PRJEB30317
ERR3263739	Human	2015	Germany	S. Typhi	PRJEB30317
ERR3263740	Human	2015	Germany	S. Enteritidis	PRJEB30317
ERR3263741	Human	2015	Germany	S. Typhi	PRJEB30317
ERR3263742	Human	2015	Germany	S. Typhi	PRJEB30317
ERR3263743	Not provided	2015	Germany	S. Typhi	PRJEB30317
ERR3263744	Human	2015	Germany	S. Typhi	PRJEB30317
ERR3263745	Human	2015	Germany	S. Typhi	PRJEB30317
ERR3263746	Human	2015	Germany	S. Typhi	PRJEB30317
ERR3263747	Human	2015	Germany	S. Typhi	PRJEB30317
ERR3263748	Human	2015	Germany	S. Enteritidis	PRJEB30317
ERR3263749	Human	2015	Germany	S. Enteritidis	PRJEB30317
ERR3263750	Human	2015	Germany	S. Enteritidis	PRJEB30317
ERR3263751	Human	2015	Germany	S. Enteritidis	PRJEB30317
ERR3263752	Human	2015	Germany	S. Enteritidis	PRJEB30317
ERR3263753	Human	2015	Germany	S. Enteritidis	PRJEB30317
ERR3263754	Human	2015	Germany	S. Enteritidis	PRJEB30317
ERR3263755	Human	2015	Germany	S. Typhi	PRJEB30317
ERR3263756	Human	2015	Germany	S. Enteritidis	PRJEB30317
ERR3263757	Human	2015	Germany	S. Stourbridge	PRJEB30317
ERR3263758	Human	2015	Germany	S. Enteritidis	PRJEB30317
ERR3263759	Human	2015	Germany	S. Enteritidis	PRJEB30317
ERR3263760	Human	2015	Germany	S. Enteritidis	PRJEB30317
ERR3263761	Human	2015	Germany	S. Enteritidis	PRJEB30317
ERR3263762	Human	2015	Germany	S. Typhi	PRJEB30317
ERR3263763	Human	2015	Germany	S. Enteritidis	PRJEB30317
ERR3263764	Human	2015	Germany	S. Typhi	PRJEB30317
ERR3263765	Human	2015	Germany	S. Enteritidis	PRJEB30317
ERR3263766	Human	2015	Germany	S. Enteritidis	PRJEB30317
ERR3263767	Human	2015	Germany	S. Enteritidis	PRJEB30317
ERR3263768	Human	2015	Germany	S. Enteritidis	PRJEB30317
ERR3263769	Human	2015	Germany	S. Enteritidis	PRJEB30317
ERR3263770	Human	2015	Germany	S. Enteritidis	PRJEB30317
ERR3263771	Human	2015	Germany	S. Enteritidis	PRJEB30317
ERR3263772	Human	2015	Germany	S. Enteritidis	PRJEB30317
ERR3263773	Human	2015	Germany	S. Enteritidis	PRJEB30317
ERR3263774	Human	2015	Germany	S. Enteritidis	PRJEB30317
ERR3263775	Human	2015	Germany	S. Enteritidis	PRJEB30317
ERR3263776	Human	2015	Germany	S. Enteritidis	PRJEB30317
ERR3263777	Human	2015	Germany	S. Enteritidis	PRJEB30317
ERR3263778	Human	2015	Germany	S. Enteritidis	PRJEB30317
ERR3263779	Human	2015	Germany	S. Enteritidis	PRJEB30317
ERR3263780	Poultry	2015	Germany	S. Enteritidis	PRJEB30317
ERR3263781	Poultry	2015	Germany	S. Enteritidis	PRJEB30317
ERR3263782	Human	2015	Germany	S. Enteritidis	PRJEB30317
ERR3263783	Layer eggs	2015	Germany	S. Enteritidis	PRJEB30317
ERR3263784	Human	2015	Germany	S. Enteritidis	PRJEB30317
ERR3263785	Human	2015	Germany	S. Typhi	PRJEB30317
ERR3263786	Human	2015	Germany	S. Paratyphi B	PRJEB30317
ERR3263787	Human	2015	Germany	S. Typhi	PRJEB30317
ERR3263788	Human	2015	Germany	S. Enteritidis	PRJEB30317
ERR3263789	Human	2015	Germany	S. Paratyphi B	PRJEB30317
ERR3263790	Human	2015	Germany	S. Typhi	PRJEB30317
ERR3263791	Human	2015	Germany	S. Typhi	PRJEB30317
ERR3263792	Human	2015	Germany	S. Typhi	PRJEB30317
ERR3263793	Human	2015	Germany	S. Infantis	PRJEB30317
ERR3263794	Human	2015	Germany	S. Paratyphi B	PRJEB30317
ERR3263795	Human	2015	Germany	S. Typhi	PRJEB30317
ERR3263796	Human	2015	Germany	S. Typhi	PRJEB30317
ERR3263797	Human	2015	Germany	S. Typhi	PRJEB30317
ERR3263798	Human	2015	Germany	S. Typhi	PRJEB30317
ERR3263799	Human	2015	Germany	S. Typhi	PRJEB30317
ERR3263800	Human	2015	Germany	S. Typhi	PRJEB30317
ERR3263801	Human	2015	Germany	S. Typhi	PRJEB30317
ERR3263802	Human	2015	Germany	S. Typhi	PRJEB30317
ERR3263803	Human	2015	Germany	S. Typhi	PRJEB30317
ERR3263804	Human	2015	Germany	S. Typhi	PRJEB30317
ERR3263805	Human	2015	Germany	S. Typhi	PRJEB30317
ERR3263806	Human	2015	Germany	S. Stourbridge	PRJEB30317
ERR3263807	Human	2015	Germany	S. Typhi	PRJEB30317
ERR3263808	Human	2015	Germany	S. Typhi	PRJEB30317
ERR3263809	Human	2015	Germany	S. Enteritidis	PRJEB30317
ERR3263810	Human	2015	Germany	S. Infantis	PRJEB30317
ERR3263811	Human	2015	Germany	S. Infantis	PRJEB30317
ERR3263812	Human	2015	Germany	S. Infantis	PRJEB30317
ERR3263813	Human	2016	Germany	S. Typhi	PRJEB30317
ERR3263814	Human	2018	Germany	S. Typhimurium	PRJEB30317
ERR3263815	Human	2016	Germany	S. Mbandaka	PRJEB30317
ERR3263816	Poultry	2016	Germany	S. Infantis	PRJEB30317
ERR3263817	Human	2016	Germany	S. Paratyphi B	PRJEB30317
ERR3263818	Human	2016	Germany	S. Mbandaka	PRJEB30317
ERR3263819	Human	2016	Germany	S. Typhi	PRJEB30317
ERR3263820	Human	2016	Germany	S. Typhi	PRJEB30317
ERR3263821	Human	2016	Germany	S. Typhi	PRJEB30317
ERR3263822	Human	2016	Germany	S. Infantis	PRJEB30317
ERR3263823	Human	2016	Germany	S. Infantis	PRJEB30317
ERR3263824	Human	2016	Germany	S. Infantis	PRJEB30317
ERR3263825	Human	2016	Germany	S. Infantis	PRJEB30317
ERR3263826	Human	2016	Germany	S. Infantis	PRJEB30317
ERR3263827	Cattle	2016	Germany	S. Infantis	PRJEB30317
ERR3263828	Cattle	2016	Germany	S. Infantis	PRJEB30317
ERR3263829	Human	2016	Germany	S. Typhi	PRJEB30317
ERR3263830	Human	2016	Germany	S. Infantis	PRJEB30317
ERR3263831	Pigeon	2016	Germany	S. Infantis	PRJEB30317
ERR3263832	Human	2016	Germany	S. Infantis	PRJEB30317
ERR3263833	Human	2016	Germany	S. Mbandaka	PRJEB30317
ERR3263834	Human	2016	Germany	S. Paratyphi B	PRJEB30317
ERR3263835	Human	2016	Germany	S. Typhi	PRJEB30317
ERR3263836	Human	2016	Germany	S. Choleraesuis	PRJEB30317
ERR3263837	Human	2016	Germany	S. Choleraesuis	PRJEB30317
ERR3263838	Human	2016	Germany	S. Typhi	PRJEB30317
ERR3263839	Human	2016	Germany	S. Enteritidis	PRJEB30317
ERR3263840	Feed	2016	Germany	S. Mbandaka	PRJEB30317
ERR3263841	Human	2016	Germany	S. Enteritidis	PRJEB30317
ERR3263842	Human	2016	Germany	S. Enteritidis	PRJEB30317
ERR3263843	Human	2016	Germany	S. Mbandaka	PRJEB30317
ERR3263844	Human	2016	Germany	S. Mbandaka	PRJEB30317
ERR3263845	Human	2016	Germany	S. Mbandaka	PRJEB30317
ERR3263846	Human	2016	Germany	S. Mbandaka	PRJEB30317
ERR3263847	Human	2016	Germany	S. Mbandaka	PRJEB30317
ERR3263848	Human	2016	Germany	S. Mbandaka	PRJEB30317
ERR3263849	Human	2016	Germany	S. Mbandaka	PRJEB30317
ERR3263850	Human	2016	Germany	S. Mbandaka	PRJEB30317
ERR3263851	Human	2016	Germany	S. Mbandaka	PRJEB30317
ERR3263852	Human	2016	Germany	S. Mbandaka	PRJEB30317
ERR3263853	Human	2016	Germany	S. Mbandaka	PRJEB30317
ERR3263854	Human	2016	Germany	S. Enteritidis	PRJEB30317
ERR3263855	Human	2016	Germany	S. Enteritidis	PRJEB30317
ERR3263856	Layer eggs	2016	Germany	S. Enteritidis	PRJEB30317
ERR3263857	Human	2016	Germany	S. Enteritidis	PRJEB30317
ERR3263858	Human	2016	Germany	S. Typhi	PRJEB30317
ERR3263859	Human	2016	Germany	S. Typhi	PRJEB30317
ERR3263860	Human	2016	Germany	S. Enteritidis	PRJEB30317
ERR3263861	Human	2016	Germany	S. Typhi	PRJEB30317
ERR3263862	Human	2016	Germany	S. Typhi	PRJEB30317
ERR3263863	Human	2016	Germany	S. Choleraesuis	PRJEB30317
ERR3263864	Human	2016	Germany	S. Typhi	PRJEB30317
ERR3263865	Human	2016	Germany	S. Enteritidis	PRJEB30317
ERR3263866	Human	2016	Germany	S. Choleraesuis	PRJEB30317
ERR3263867	Human	2016	Germany	S. Enteritidis	PRJEB30317
ERR3263868	Human	2016	Germany	S. Paratyphi B	PRJEB30317
ERR3263869	Human	2016	Germany	S. Typhimurium	PRJEB30317
ERR3263870	Human	2016	Germany	S. Enteritidis	PRJEB30317
ERR3263871	Human	2016	Germany	S. Enteritidis	PRJEB30317
ERR3263872	Human	2016	Germany	S. Enteritidis	PRJEB30317
ERR3263873	Human	2016	Germany	S. Enteritidis	PRJEB30317
ERR3263874	Human	2016	Germany	S. Enteritidis	PRJEB30317
ERR3263875	Human	2016	Germany	S. ssp.I serol. Rough	PRJEB30317
ERR3263876	Human	2016	Germany	S. Enteritidis	PRJEB30317
ERR3263877	Human	2016	Germany	S. Typhi	PRJEB30317
ERR3263878	Human	2016	Germany	S. Typhi	PRJEB30317
ERR3263879	Human	2016	Germany	S. Typhi	PRJEB30317
ERR3263880	Human	2016	Germany	S. ssp.I serol. Rough	PRJEB30317
ERR3263881	Human	2016	Germany	S. Choleraesuis	PRJEB30317
ERR3263882	Human	2016	Germany	S. Enteritidis	PRJEB30317
ERR3263883	Human	2016	Germany	S. Enteritidis	PRJEB30317
ERR3263884	Human	2016	Germany	S. Enteritidis	PRJEB30317
ERR3263885	Human	2016	Germany	S. Enteritidis	PRJEB30317
ERR3263886	Human	2016	Germany	S. Enteritidis	PRJEB30317
ERR3263887	Human	2016	Germany	S. Typhi	PRJEB30317
ERR3263888	Not proviede	2016	Germany	S. Typhi	PRJEB30317
ERR3263889	Human	2016	Germany	S. ssp. serol. Rough	PRJEB30317
ERR3263890	Human	2016	Germany	S. Infantis	PRJEB30317
ERR3263891	Human	2016	Germany	S. Enterititdis	PRJEB30317
ERR3263892	Human	2016	Germany	S. Typhimurium	PRJEB30317
ERR3263893	Poultry	2016	Germany	S. ssp.I serol. Rough	PRJEB30317
ERR3263894	Human	2016	Germany	S. ssp.II serol. Rough	PRJEB30317
ERR3263895	Human	2016	Germany	S. Typhimurium	PRJEB30317
ERR3263896	Human	2016	Germany	S. Enteritidis	PRJEB30317
ERR3263897	Human	2016	Germany	S. Enteritidis	PRJEB30317
ERR3263898	Human	2016	Germany	S. Enteritidis	PRJEB30317
ERR3263899	Human	2016	Germany	S. Enteritidis	PRJEB30317
ERR3263900	Human	2016	Germany	S. Enteritidis	PRJEB30317
ERR3263901	Human	2016	Germany	S. Paratyphi B	PRJEB30317
ERR3263902	Human	2016	Germany	S. Enteritidis	PRJEB30317
ERR3263903	Human	2016	Germany	S. Typhimurium	PRJEB30317
ERR3263904	Human	2016	Germany	S. Enteritidis	PRJEB30317
ERR3263905	Human	2016	Germany	S. Enteritidis	PRJEB30317
ERR3263906	Human	2016	Germany	S. Typhimurium	PRJEB30317
ERR3263907	Human	2016	Germany	S. Stourbridge	PRJEB30317
ERR3263908	Human	2016	Germany	S. Enteritidis	PRJEB30317
ERR3263909	Human	2016	Germany	S. Enteritidis	PRJEB30317
ERR3263910	Human	2016	Germany	S. Stourbridge	PRJEB30317
ERR3263911	Human	2016	Germany	S. Stourbridge	PRJEB30317
ERR3263912	Human	2016	Germany	S. Typhimurium	PRJEB30317
ERR3263913	Human	2016	Germany	S. Enteritidis	PRJEB30317
ERR3263914	Human	2016	Germany	S. Enteritidis	PRJEB30317
ERR3263915	Human	2016	Germany	S. Typhi	PRJEB30317
ERR3263916	Human	2016	Germany	S. Enteritidis	PRJEB30317
ERR3263917	Human	2016	Germany	S. Typhimurium	PRJEB30317
ERR3263918	Human	2016	Germany	S. Typhimurium	PRJEB30317
ERR3263919	Human	2016	Germany	S. Enteritidis	PRJEB30317
ERR3263920	Human	2016	Germany	S. Enteritidis	PRJEB30317
ERR3263921	Human	2016	Germany	S. Enteritidis	PRJEB30317
ERR3263922	Human	2016	Germany	S. Enteritidis	PRJEB30317
ERR3263923	Human	2016	Germany	S. Typhimurium	PRJEB30317
ERR3263924	Human	2016	Germany	S. Typhimurium	PRJEB30317
ERR3263925	Human	2016	Germany	S. Enteritidis	PRJEB30317
ERR3263926	Human	2016	Germany	S. Typhimurium	PRJEB30317
ERR3263927	Human	2016	Germany	S. Typhimurium	PRJEB30317
ERR3263928	Human	2016	Germany	S. Enteritidis	PRJEB30317
ERR3263929	Human	2016	Germany	S. Typhi	PRJEB30317
ERR3263930	Human	2016	Germany	S. Enteritidis	PRJEB30317
ERR3263931	Human	2016	Germany	S. Enteritidis	PRJEB30317
ERR3263932	Human	2016	Germany	S. Enteritidis	PRJEB30317
ERR3263933	Human	2016	Germany	S. Enteritidis	PRJEB30317
ERR3263934	Human	2016	Germany	S. Enteritidis	PRJEB30317
ERR3263935	Human	2016	Germany	S. Enteritidis	PRJEB30317
ERR3263936	Human	2016	Germany	S. Enteritidis	PRJEB30317
ERR3263937	Human	2016	Germany	S. Infantis	PRJEB30317
ERR3263938	Human	2016	Germany	S. Stourbridge	PRJEB30317
ERR3263939	Human	2016	Germany	S. Enteritidis	PRJEB30317
ERR3263940	Human	2016	Germany	S. Enteritidis	PRJEB30317
ERR3263941	Human	2016	Germany	S. Enteritidis	PRJEB30317
ERR3263942	Human	2016	Germany	S. Enteritidis	PRJEB30317
ERR3263943	Human	2016	Germany	S. Enteritidis	PRJEB30317
ERR3263944	Human	2016	Germany	S. Enteritidis	PRJEB30317
ERR3263945	Human	2016	Germany	S. Enteritidis	PRJEB30317
ERR3263946	Human	2016	Germany	S. Infantis	PRJEB30317
ERR3263947	Human	2016	Germany	S. Infantis	PRJEB30317
ERR3263948	Human	2016	Germany	S. Typhimurium	PRJEB30317
ERR3263949	Human	2016	Germany	S. Typhi	PRJEB30317
ERR3263950	Human	2016	Germany	S. Infantis	PRJEB30317
ERR3263951	Human	2016	Germany	S. Typhi	PRJEB30317
ERR3263952	Human	2016	Germany	S. Typhi	PRJEB30317
ERR3263953	Human	2016	Germany	S. Kentucky	PRJEB30317
ERR3263954	Human	2016	Germany	S. Typhimurium	PRJEB30317
ERR3263955	Human	2016	Germany	S. Infantis	PRJEB30317
ERR3263956	Human	2016	Germany	S. Infantis	PRJEB30317
ERR3263957	Human	2016	Germany	S. Infantis	PRJEB30317
ERR3263958	Human	2016	Germany	S. Infantis	PRJEB30317
ERR3263959	Human	2016	Germany	S. Infantis	PRJEB30317
ERR3263960	Human	2016	Germany	S. Infantis	PRJEB30317
ERR3263961	Human	2016	Germany	S. Stourbridge	PRJEB30317
ERR3263962	Human	2016	Germany	S. Typhi	PRJEB30317
ERR3263963	Human	2016	Germany	S. Stourbridge	PRJEB30317
ERR3263964	Human	2016	Germany	S. Typhi	PRJEB30317
ERR3263965	Human	2016	Germany	S. Infantis	PRJEB30317
ERR3263966	Human	2016	Germany	S. Infantis	PRJEB30317
ERR3263967	Human	2016	Germany	S. Infantis	PRJEB30317
ERR3263968	Human	2016	Germany	S. Infantis	PRJEB30317
ERR3263969	Human	2016	Germany	S. Infantis	PRJEB30317
ERR3263970	Human	2016	Germany	S. Infantis	PRJEB30317
ERR3263971	Human	2016	Germany	S. Stourbridge	PRJEB30317
ERR3263972	Human	2016	Germany	S. Typhimurium	PRJEB30317
ERR3263973	Human	2016	Germany	S. Typhimurium	PRJEB30317
ERR3263974	Human	2016	Germany	S. Infantis	PRJEB30317
ERR3263975	Human	2016	Germany	S. Typhi	PRJEB30317
ERR3263976	Human	2016	Germany	S. Typhi	PRJEB30317
ERR3263977	Human	2016	Germany	S. Typhimurium	PRJEB30317
ERR3263978	Human	2016	Germany	S. Stourbridge	PRJEB30317
ERR3263979	Human	2016	Germany	S. Typhimurium	PRJEB30317
ERR3263980	Human	2016	Germany	S. Kentucky	PRJEB30317
ERR3263981	Human	2016	Germany	S. Choleraesuis	PRJEB30317
ERR3263982	Human	2016	Germany	S. Choleraesuis	PRJEB30317
ERR3263983	Human	2016	Germany	S. Choleraesuis	PRJEB30317
ERR3263984	Human	2016	Germany	S. Choleraesuis	PRJEB30317
ERR3263985	Human	2016	Germany	S. Choleraesuis	PRJEB30317
ERR3263986	Human	2016	Germany	S. enterica 11:z41:e,n,z15	PRJEB30317
ERR3263987	Human	2016	Germany	S. Typhi	PRJEB30317
ERR3263988	Human	2016	Germany	S. Typhi	PRJEB30317
ERR3263989	Human	2016	Germany	S. Choleraesuis	PRJEB30317
ERR3263990	Human	2016	Germany	S. Infantis	PRJEB30317
ERR3263991	Human	2016	Germany	S. Infantis	PRJEB30317
ERR3263992	Human	2016	Germany	S. Infantis	PRJEB30317
ERR3263993	Human	2016	Germany	S. Typhi	PRJEB30317
ERR3263994	Human	2016	Germany	S. Typhi	PRJEB30317
ERR3263995	Human	2016	Germany	S. Typhi	PRJEB30317
ERR3263996	Human	2016	Germany	S. Choleraesuis	PRJEB30317
ERR3263997	Human	2016	Germany	S. Choleraesuis	PRJEB30317
ERR3263998	Human	2016	Germany	S. Choleraesuis	PRJEB30317
ERR3263999	Human	2016	Germany	S. Choleraesuis	PRJEB30317
ERR3264000	Human	2016	Germany	S. Typhi	PRJEB30317
ERR3264001	Human	2016	Germany	S. Choleraesuis var. Kunzendorf (mono)	PRJEB30317
ERR3264002	Human	2016	Germany	S. Enteritidis	PRJEB30317
ERR3264003	Human	2016	Germany	S. Typhimurium	PRJEB30317
ERR3264004	Human	2016	Germany	S. Choleraesuis	PRJEB30317
ERR3264005	Human	2016	Germany	S. Typhimurium	PRJEB30317
ERR3264006	Human	2016	Germany	S. Typhimurium	PRJEB30317
ERR3264007	Human	2016	Germany	S. Typhimurium	PRJEB30317
ERR3264008	Human	2016	Germany	S. Typhimurium	PRJEB30317
ERR3264009	Human	2016	Germany	S. Choleraesuis	PRJEB30317
ERR3264010	Human	2018	Germany	S. Typhi	PRJEB30317
ERR3264011	Human	2016	Germany	S. enterica 11:z41:e,n,z15	PRJEB30317
ERR3264012	Human	2016	Germany	S. Typhi	PRJEB30317
ERR3264013	Human	2016	Germany	S. Choleraesuis	PRJEB30317
ERR3264014	Human	2016	Germany	S. Choleraesuis	PRJEB30317
ERR3264015	Human	2016	Germany	S. Choleraesuis	PRJEB30317
ERR3264016	Human	2017	Germany	S. enterica 11:z41:e,n,z15	PRJEB30317
ERR3264017	Human	2017	Germany	S. enterica 11:z41:e,n,z15	PRJEB30317
ERR3264018	Human	2017	Germany	S. enterica 11:z41:e,n,z15	PRJEB30317
ERR3264019	Human	2017	Germany	S. Choleraesuis	PRJEB30317
ERR3264020	Human	2017	Germany	S. Choleraesuis	PRJEB30317
ERR3264022	Human	2017	Germany	S. Paratyphi C	PRJEB30317
ERR3264023	Human	2017	Germany	S. Choleraesuis	PRJEB30317
ERR3264024	Human	2017	Germany	S. enterica 11:z41:e,n,z15	PRJEB30317
ERR3264025	Human	2017	Germany	S. Choleraesuis	PRJEB30317
ERR3264026	Human	2017	Germany	S. Choleraesuis var. Kunzendorf (mono)	PRJEB30317
ERR3264027	Human	2017	Germany	S. Stourbridge	PRJEB30317
ERR3264028	Human	2017	Germany	S. Stourbridge	PRJEB30317
ERR3264029	Human	2017	Germany	S. enterica 11:z41:e,n,z15	PRJEB30317
ERR3264030	Human	2017	Germany	S. Choleraesuis	PRJEB30317
ERR3264031	Human	2017	Germany	S. enterica 11:z41:e,n,z15	PRJEB30317
ERR3264032	Human	2017	Germany	S. Stourbridge	PRJEB30317
ERR3264033	Human	2017	Germany	S. Choleraesuis	PRJEB30317
ERR3264034	Human	2017	Germany	S. Paratyphi C	PRJEB30317
ERR3264035	Human	2017	Germany	S. Choleraesuis var. Kunzendorf (mono)	PRJEB30317
ERR3264036	Human	2017	Germany	S. ssp. serol. Rough	PRJEB30317
ERR3264037	Human	2017	Germany	S. enterica 11:z41:e,n,z15	PRJEB30317
ERR3264038	Human	2017	Germany	S. Choleraesuis	PRJEB30317
ERR3264039	Human	2017	Germany	S. Stourbridge	PRJEB30317
ERR3264040	Human	2017	Germany	S. enterica 11:z41:e,n,z15	PRJEB30317
ERR3264041	Human	2017	Germany	S. Kentucky	PRJEB30317
ERR3264042	Human	2017	Germany	S. Kentucky	PRJEB30317
ERR3264043	Human	2017	Germany	S. Kentucky	PRJEB30317
ERR3264044	Human	2017	Germany	S. Kottbus	PRJEB30317
ERR3264045	Human	2017	Germany	S. Kottbus	PRJEB30317
ERR3264046	Human	2017	Germany	S. Kottbus	PRJEB30317
ERR3264047	Human	2017	Germany	S. Kottbus	PRJEB30317
ERR3264048	Human	2017	Germany	S. Kottbus	PRJEB30317
ERR3264049	Human	2017	Germany	S. Kottbus	PRJEB30317
ERR3264050	Human	2017	Germany	S. Kottbus	PRJEB30317
ERR3264051	Human	2017	Germany	S. Kottbus	PRJEB30317
ERR3264052	Human	2017	Germany	S. Kottbus	PRJEB30317
ERR3264053	Human	2017	Germany	S. Kottbus	PRJEB30317
ERR3264054	Human	2017	Germany	S. Kottbus	PRJEB30317
ERR3264055	Human	2017	Germany	S. Kentucky	PRJEB30317
ERR3264056	Human	2017	Germany	S. Kottbus	PRJEB30317
ERR3264057	Human	2017	Germany	S. Agona	PRJEB30317
ERR3264058	Human	2017	Germany	S. Choleraesuis	PRJEB30317
ERR3264059	Human	2017	Germany	S. Agona	PRJEB30317
ERR3264060	Human	2017	Germany	S. Kentucky	PRJEB30317
ERR3264062	Human	2018	Germany	S. Enteritidis	PRJEB30317
ERR3264063	Human	2018	Germany	S. Choleraesuis	PRJEB30317
ERR3264064	Human	2018	Germany	S. Choleraesuis	PRJEB30317
ERR3264065	Human	2018	Germany	S. Choleraesuis	PRJEB30317
ERR3264066	Human	2018	Germany	S. Choleraesuis	PRJEB30317
ERR3264067	Human	2018	Germany	S. Choleraesuis	PRJEB30317
ERR3264068	Human	2018	Germany	S. Agona	PRJEB30317
ERR3264069	Human	2018	Germany	S. Derby	PRJEB30317
ERR3264070	Human	2018	Germany	S. Derby	PRJEB30317
ERR3264071	Human	2018	Germany	S. Derby	PRJEB30317
ERR3264072	Human	2018	Germany	S. Derby	PRJEB30317
ERR3264073	Human	2018	Germany	S. Derby	PRJEB30317
ERR3264074	Human	2017	Germany	S. Enteritidis	PRJEB30317
ERR3264075	Human	2018	Germany	S. Infantis	PRJEB30317
ERR3264076	Human	2018	Germany	S. Infantis	PRJEB30317
ERR3264077	Human	2018	Germany	S. Infantis	PRJEB30317
ERR3264078	Human	2018	Germany	S. Infantis	PRJEB30317
ERR3264079	Human	2018	Germany	S. Infantis	PRJEB30317
ERR3264080	Human	2018	Germany	S. Mikawasima	PRJEB30317
ERR3264081	Human	2018	Germany	S. Mikawasima	PRJEB30317
ERR3264082	Human	2018	Germany	S. Mikawasima	PRJEB30317
ERR3264083	Human	2018	Germany	S. Mikawasima	PRJEB30317
ERR3264084	Human	2018	Germany	S. Mikawasima	PRJEB30317
ERR3264085	Human	2018	Germany	S. Mikawasima	PRJEB30317
ERR3264086	Human	2018	Germany	S. Mikawasima	PRJEB30317
ERR3264087	Human	2018	Germany	S. Mikawasima	PRJEB30317
ERR3264088	Human	2018	Germany	S. Mikawasima	PRJEB30317
ERR3264089	Human	2018	Germany	S. Typhimurium	PRJEB30317
ERR3264090	Human	2018	Germany	S. Mikawasima	PRJEB30317
ERR3264091	Human	2018	Germany	S. Infantis	PRJEB30317
ERR3264092	Human	2018	Germany	S. Infantis	PRJEB30317
ERR3264021	Human	2018	Germany	S. Strathcona	PRJEB30317
ERR3264061	Human	2018	Germany	S. Strathcona	PRJEB30317
ERR2003327	Human	2000	Germany	S. Typhimurium mono	PRJEB16326
ERR2003328	Human	2001	Germany	S. Typhimurium mono	PRJEB16326
ERR2003329	Human	2001	Germany	S. Typhimurium mono	PRJEB16326
ERR2003330	Pigs	2002	Germany	S. Typhimurium mono	PRJEB16326
ERR2003331	Human	2002	Germany	S. Typhimurium mono	PRJEB16326
ERR2003332	Pigs	2003	Germany	S. Typhimurium mono	PRJEB16326
ERR2003341	Human	2004	Germany	S. Typhimurium	PRJEB16326
ERR2003333	Pigs	2004	Germany	S. Typhimurium mono	PRJEB16326
ERR2003334	Human	2004	Germany	S. Typhimurium mono	PRJEB16326
ERR2003335	Cattle	2005	Germany	S. Typhimurium mono	PRJEB16326
ERR2099818	Human	2016	Germany	S. Typhimurium	PRJEB16326
ERR2099823	Human	2016	Germany	S. Typhimurium	PRJEB16326
ERR2099820	Human	2016	Germany	S. Typhimurium	PRJEB16326
ERR2099822	Pigs	2016	Germany	S. Typhimurium	PRJEB16326
ERR1912198	Human	2016	Germany	S. Typhimurium	PRJEB16326
ERR2003365	Human	2016	Germany	S. Typhimurium	PRJEB16326
ERR2003364	Human	2016	Germany	S. Typhimurium mono	PRJEB16326
ERR2003363	Human	2016	Germany	S. Typhimurium	PRJEB16326
ERR2003362	Pigs	2016	Germany	S. Typhimurium mono	PRJEB16326
ERR2003361	Human	2016	Germany	S. Typhimurium mono	PRJEB16326
ERR2003360	Human	2016	Germany	S. Typhimurium mono	PRJEB16326
ERR2003359	Human	2016	Germany	S. Typhimurium	PRJEB16326
ERR2003358	Human	2016	Germany	S. Typhimurium	PRJEB16326
ERR2003357	Human	2016	Germany	S. Typhimurium	PRJEB16326
ERR2003356	Human	2016	Germany	S. Typhimurium	PRJEB16326
ERR2003355	Human	2016	Germany	S. Typhimurium	PRJEB16326
ERR2003354	Human	2016	Germany	S. Typhimurium	PRJEB16326
ERR2003353	Human	2016	Germany	S. Typhimurium mono	PRJEB16326
ERR2003352	Human	2016	Germany	S. Typhimurium	PRJEB16326
ERR2003351	Human	2016	Germany	S. Typhimurium mono	PRJEB16326
ERR2003350	Human	2016	Germany	S. Typhimurium mono	PRJEB16326
ERR2003349	Human	2016	Germany	S. Typhimurium mono	PRJEB16326
ERR2003348	Human	2016	Germany	S. Typhimurium	PRJEB16326
ERR2003347	Human	2016	Germany	S. Typhimurium mono	PRJEB16326
ERR2003346	Human	2016	Germany	S. Typhimurium	PRJEB16326
ERR2003345	Human	2016	Germany	S. Typhimurium mono	PRJEB16326
ERR2003344	Human	2016	Germany	S. Typhimurium	PRJEB16326
ERR2003343	Human	2016	Germany	S. Typhimurium	PRJEB16326
ERR2003342	Human	2016	Germany	S. Typhimurium mono	PRJEB16326
ERR1950104	Human	2016	Germany	S. Typhimurium mono	PRJEB16326
ERR2003340	Human	1997	Germany	S. Typhimurium	PRJEB16326
ERR2003336	Human	1997	Germany	S. Typhimurium	PRJEB16326
ERR2003337	Human	1997	Germany	S. Typhimurium	PRJEB16326
ERR2003338	Human	1997	Germany	S. Typhimurium	PRJEB16326
ERR2003339	Human	1997	Germany	S. Typhimurium	PRJEB16326

The dataset consists of 520 whole genome sequences, which have been submitted to ENA.

