# Supplementary material for: Genome-based Salmonella serotyping as the new gold standard
Source: Sci Rep. 2020 Mar 9;10:4333. doi: 10.1038/s41598-020-61254-1 (PMC7062728; doi:10.1038/s41598-020-61254-1)
Supplement: Supplementary file 1 — Supplementary information . [file 41598_2020_61254_MOESM1_ESM.pdf]

Supplementary Information

Genome-based *Salmonella* serotyping as the new gold standard

Sangeeta Banerji, Sandra Simon, Andreas Tille, Angelika Fruth and Antje Flieger

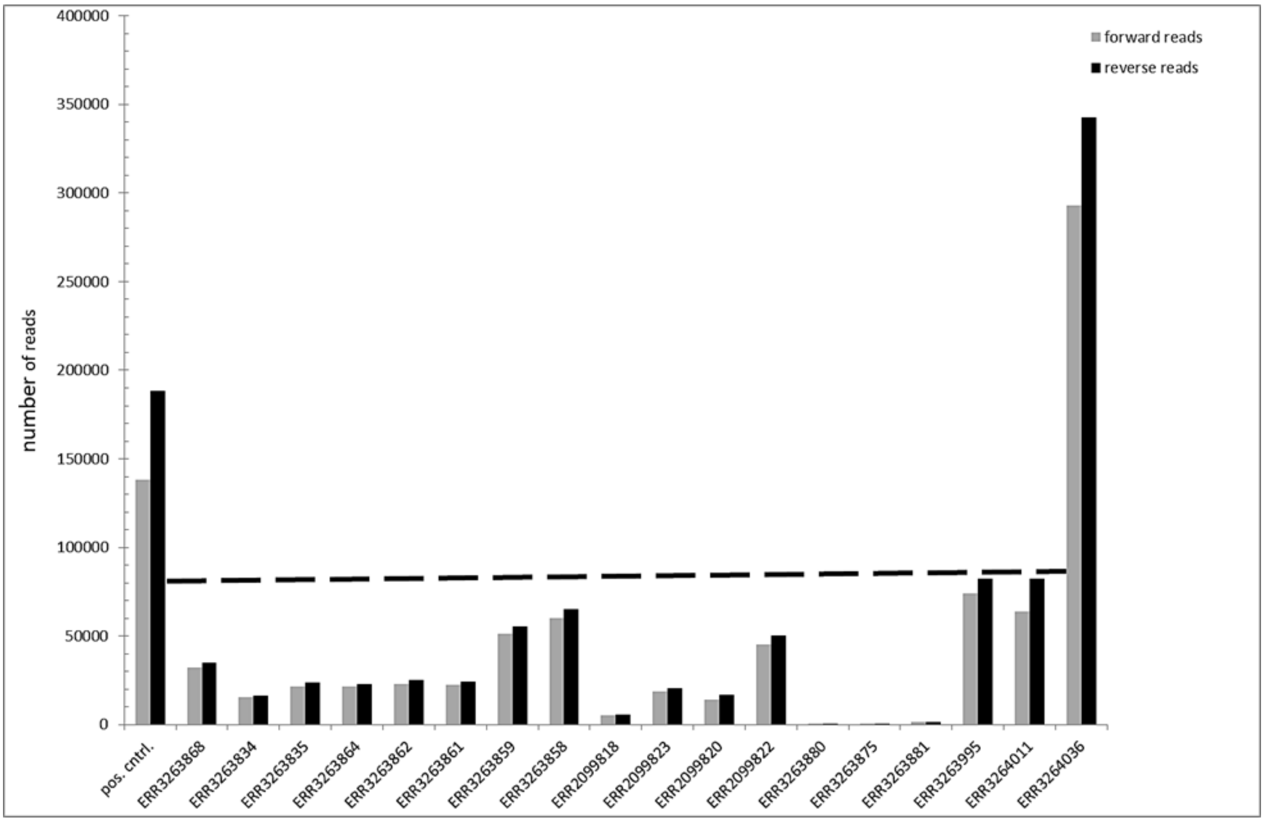

Fig. S1. Overview of data quality of sequences with failed serotype prediction by SeqSero. Data quality is represented by the number of reads in the forward and reverse read files of paired-end reads. Isolates with unsuccessful serotype prediction by SeqSero are shown as well as a positive control (ERR3263813) with successful serotype prediction. Please note that for isolate ERR3264036 serotype prediction failed in spite of sufficient data quality because of missing O-7 antigen locus (Fig. S3). The dashed line indicates the threshold of 80,000 reads per direction for paired end reads to achieve a minimal theoretical coverage of 10-fold.

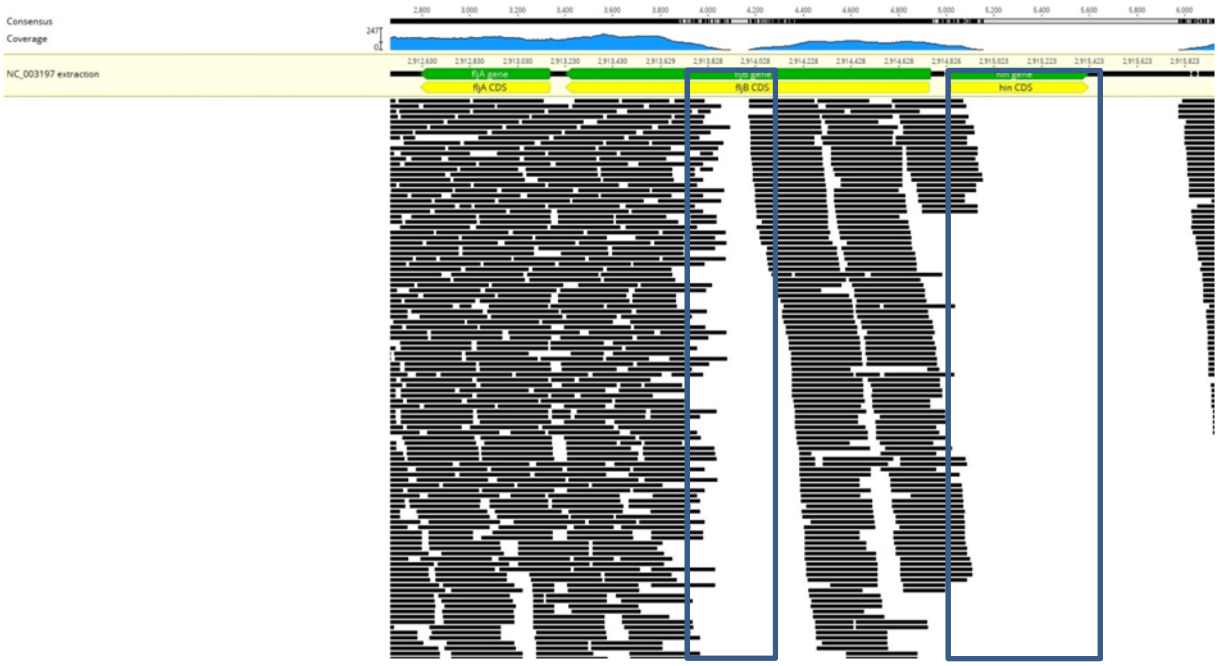

Fig. S2. Isolate ERR2003330 lacks the invertase gene *hin* as well as the central part of the *fljB* gene. Raw reads of Isolate ERR2003330 were mapped against the conserved *fljAB* region extracted from *S.* Typhimurium LT2 using the Geneious mapper in Geneious.

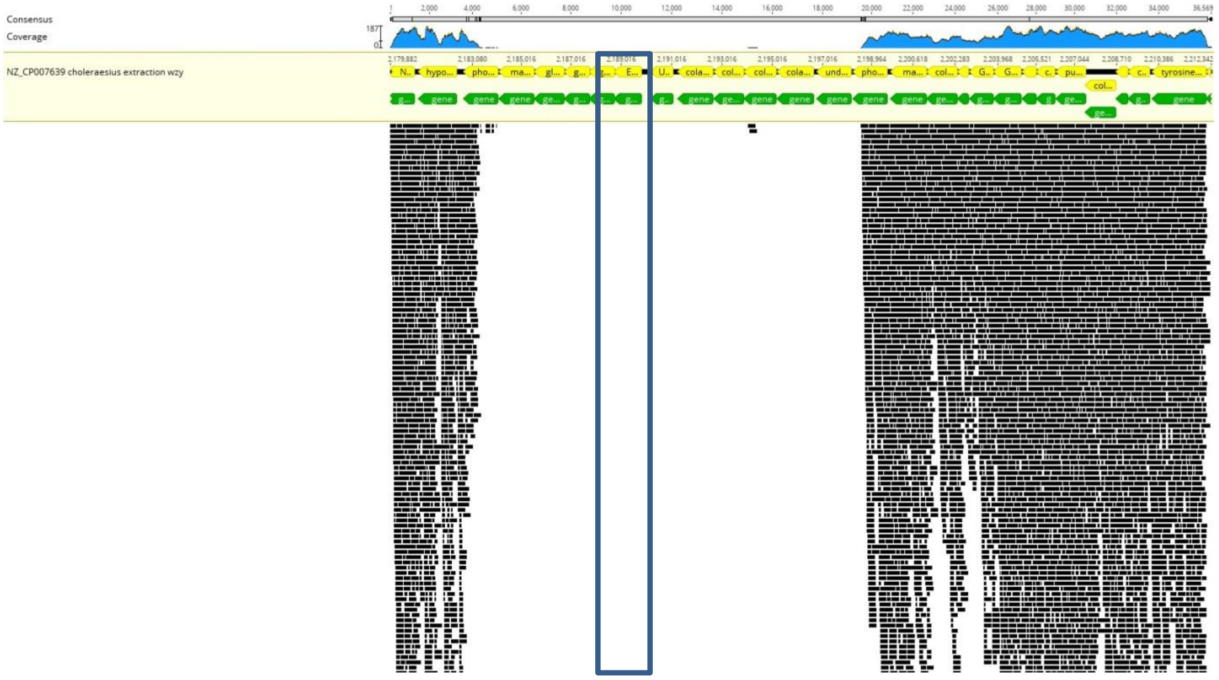

Fig. S3. *S.* Choleraesuis O-7 antigen locus and surrounding region are missing in isolate ERR3264036. Raw reads of isolate ERR3264036 were mapped against region EL48_RS10955- EL48_RS11010, including the *wzy* locus EL48_RS10980 of *S.* Choleraesuis NZ_CP007639 using Bowtie2 in Geneious.

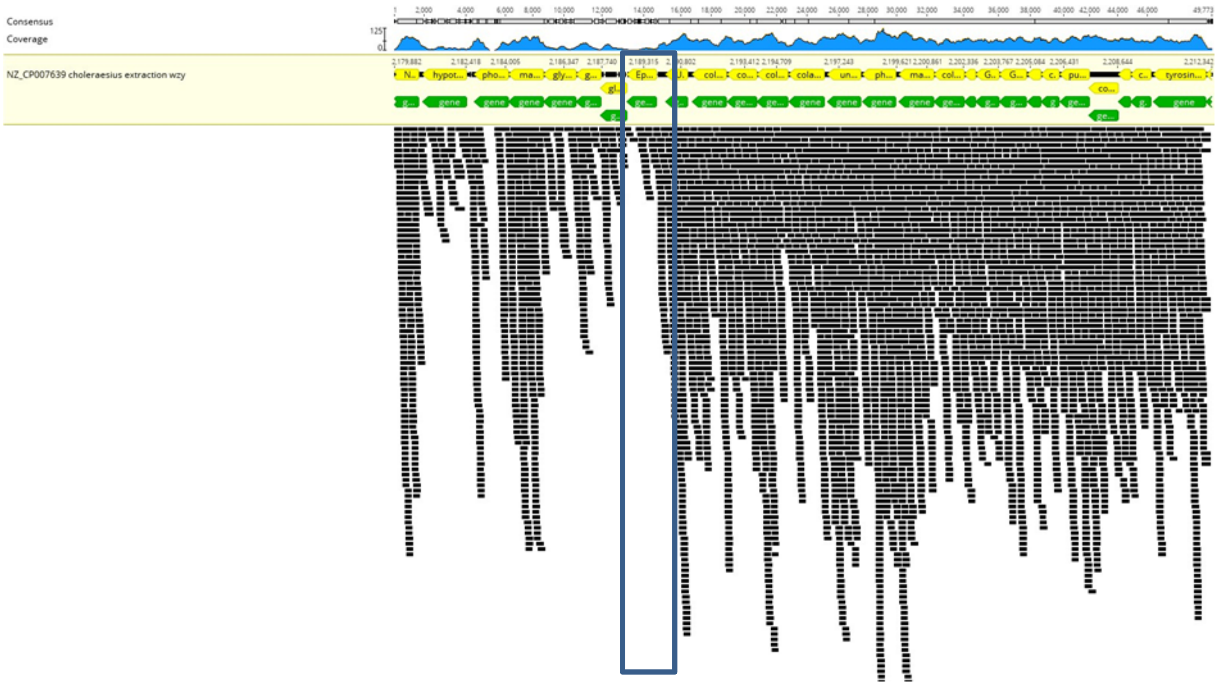

Fig. S4. Poor read coverage of *S.* Choleraesuis O-7 antigen locus in isolate ERR3264063. Raw reads of isolate ERR3264063 were mapped against region EL48_RS10955- EL48_RS11010, including the *wzy* locus EL48_RS10980 of *S.* Choleraesuis NZ_CP007639 using Bowtie2 in Geneious. This figure is also representative for isolates ERR3264067 and ERR3264066.

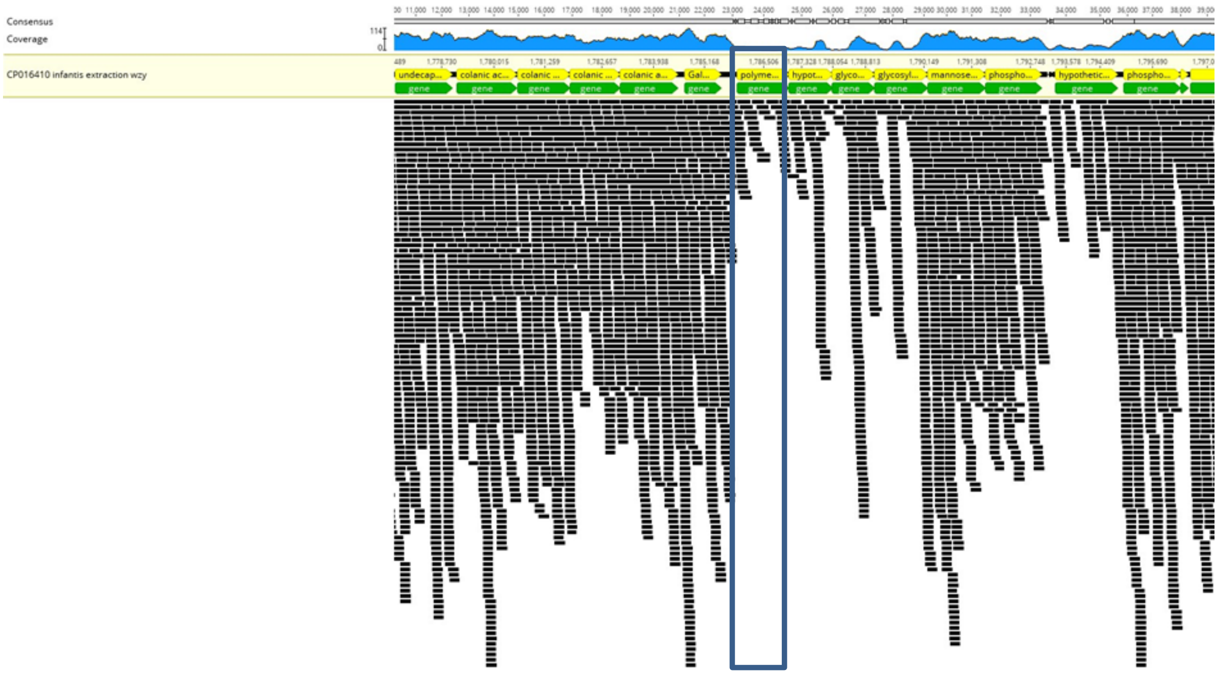

Fig. S5. Poor read coverage of *S. Infantis* O-7 antigen locus in isolate ERR3264076. Raw reads of isolate ERR3264076 were mapped against region APL92_08575- APL92_08690, including the *wzy* locus APL92_08605 of *S.* Infantis CP016410 using Bowtie2 in Geneious.
